# Genome Sequence of a Novel Porcine Circovirus Type 2 Strain That Reemerged in Southern China

**DOI:** 10.1128/genomeA.01567-16

**Published:** 2017-02-16

**Authors:** Shao-Lun Zhai, Xia Zhou, Tao Lin, He Zhang, Dian-Hong Lv, Xiao-Hui Wen, Xiu-Rong Zhou, Chun-Ling Jia, Du Tu, Xue-Liang Zhu, Qin-Ling Chen, Wen-Kang Wei

**Affiliations:** a Guangdong Key Laboratory of Animal Disease Prevention, Animal Disease Diagnostic Center, Institute of Animal Health, Guangdong Academy of Agricultural Sciences, Guangzhou, China; b Guangdong Provincial Animal Disease Diagnostic Engineering Technology Research Center, Guangzhou, China; c College of Veterinary Medicine, South China Agricultural University, Guangzhou, China; d Department of Chemistry and Biochemistry, South Dakota State University, Brookings, South Dakota, USA

## Abstract

Here, we describe a novel porcine circovirus type 2 (PCV2) variant (GD2014) found in the Guangdong province, southern China. Its complete genome is 1,766 nucleotides and contained a 708-nucleotide open reading frame 2 (ORF2). Sequence analysis suggested that GD2014 is closest to JS2015 originating from the Jiangsu province of China and belongs to the PCV2d genotype.

## GENOME ANNOUNCEMENT

Porcine circoviruses (PCVs), members of the *Circovirus* genus in the *Circoviridae* family, are divided into two known types, porcine circovirus type 1 (PCV1) and porcine circovirus type 2 (PCV2). Further genotyping analysis indicated that PCV2 had five confirmed genotypes (PCV2a, PCV2b, PCV2c, PCV2d, and PCV2e) (1, 2). In particular, PCV2 is closely related to porcine circovirus associated disease (PCVAD) and has a serious economic impact on the world swine industry (3). At present, PCV2 vaccines based on different genotypes work against PCVAD and can decrease the viral presence and viral loads of PCV2 (3, 4). However, PCV2 still occurs frequently on vaccinating and nonvaccinating swine farms (5–8). Specifically, some bizarre PCV2 strains have emerged, which are distinct from the classical PCV2 strains (3). However, they were rarely found in our survey until now. In the present study, the genome of a novel PCV2d-like variant (GD2014) was identified in the Guangdong province, southern China.

In the epidemiological survey of PCV2, we obtained the full-length nucleotide genome of a novel variant (named GD2014) according to a previous PCR method (9). Due to a guanine (G) deletion at position 1,044 compared with PCV2d, its complete genome was 1,766 nucleotides long with a G+C content of 48.36%. Moreover, based on the deletion, its open reading frame 2 (ORF2) sequences were lengthened to 708 nucleotides, which had three or six nucleotides more than that of PCV2a, PCV2b, PCV2c, and PCV2d representative strains (3). However, ORF2 sequences of GD2014 were three nucleotides shorter than those of PCV2e representative strains (1). Multiple sequence alignment analysis showed that GD2014 had the closest relationship (only four different nucleotides at positions 551, 663, 940, 1,178) to JS2015 (GenBank accession no. KT220420) that emerged in the Jiangsu province, eastern China (10). Moreover, GD2014 and JS2015 had the same genome map (ORF1, 51 → 995; ORF2, 1733 → 1026). Further, phylogenetic analysis showed that GD2014 had the closest genetic relationship to the members of the PCV2d and was clustered together with JS2015.

Previously, some PCV2 deletion strains including TZ0601 (GenBank accession no. EU257511), AH (HM038030), MDJ (HM038031), and YJ (HM038032) were confirmed in China (11). However, they were scarcely reported. This study reports a PCV2 JS2015-like strain circulating in another province of China. This suggests that the prototype virus might have a stronger ability to spread than other deletion strains. Our data provided insight into the epidemiology of PCV2 and might facilitate further study of the origin and evolution of PCV2.

### Accession number(s).

The genome sequence of GD2014 has been deposited in GenBank under the accession number KY211020.
